# Modulatory effects of perforin gene dosage on pathogen-associated blood-brain barrier (BBB) disruption

**DOI:** 10.1186/s12974-016-0673-9

**Published:** 2016-08-31

**Authors:** Robin C. Willenbring, Fang Jin, David J. Hinton, Mike Hansen, Doo-Sup Choi, Kevin D. Pavelko, Aaron J. Johnson

**Affiliations:** 1 Mayo Graduate School, Mayo Clinic, Rochester, MN USA; 2 Department of Immunology, Mayo Clinic, Rochester, MN USA; 3 Department of Neurology, Mayo Clinic, Rochester, MN USA

**Keywords:** BBB disruption, CD8 T cells, Perforin, Perforin single nucleotide variants, TMEV, PIFS

## Abstract

**Background:**

CD8 T cell-mediated blood-brain barrier (BBB) disruption is dependent on the effector molecule perforin. Human perforin has extensive single nucleotide variants (SNVs), the significance of which is not fully understood. These SNVs can result in reduced, but not ablated, perforin activity or expression. However, complete loss of perforin expression or activity results in the lethal disease familial hemophagocytic lymphohistiocytosis type 2 (FHL 2). In this study, we address the hypothesis that a single perforin allele can alter the severity of BBB disruption in vivo using a well-established model of CNS vascular permeability in C57Bl/6 mice. The results of this study provide insight into the significance of perforin SNVs in the human population.

**Methods:**

We isolated the effect a single perforin allele has on CNS vascular permeability through the use of perforin-heterozygous (perforin+/−) C57BL/6 mice in the peptide-induced fatal syndrome (PIFS) model of immune-mediated BBB disruption. Seven days following Theiler’s murine encephalomyelitis virus (TMEV) CNS infection, neuroinflammation and TMEV viral control were assessed through flow cytometric analysis and quantitative real-time PCR of the viral genome, respectively. Following immune-mediated BBB disruption, gadolinium-enhanced T1-weighted MRI, with 3D volumetric analysis, and confocal microscopy were used to define CNS vascular permeability. Finally, the open field behavior test was used to assess locomotor activity of mice following immune-mediated BBB disruption.

**Results:**

Perforin-null mice had negligible CNS vascular permeability. Perforin-WT mice have extensive CNS vascular permeability. Interestingly, perforin-heterozygous mice had an intermediate level of CNS vascular permeability as measured by both gadolinium-enhanced T1-weighted MRI and fibrinogen leakage in the brain parenchyma. Differences in BBB disruption were not a result of increased CNS immune infiltrate*.* Additionally, TMEV was controlled in a perforin dose-dependent manner. Furthermore, a single perforin allele is sufficient to induce locomotor deficit during immune-mediated BBB disruption.

**Conclusions:**

Perforin modulates BBB disruption in a dose-dependent manner. This study demonstrates a potentially advantageous role for decreased perforin expression in reducing BBB disruption. This study also provides insight into the effect SNVs in a single perforin allele could have on functional deficit in neurological disease.

## Background

Neuroinflammation is a putative mechanism in pathogen-associated blood-brain barrier (BBB) disruption. Yet, mechanisms of immune-mediated BBB disruption are not fully understood. Numerous neurological diseases including cerebral malaria (CM), viral hemorrhagic fevers (VHFs), and HIV dementia have been associated with CD8 T cells [1–13]. Additionally, our lab has defined a role for CD8 T cells and perforin in mediating BBB tight junction alterations during neuroinflammation [14–16]. Perforin is necessary for BBB disruption to occur during a central nervous system (CNS) infection in these models [15]. Furthermore, CD8 T cells have the capacity to be the sole perforin-wielding cell type to induce CNS vascular permeability [16, 17]. CD8 T cells therefore can both recognize antigen and induce BBB disruption through perforin expression.

The observation that perforin is a key regulator of BBB disruption is intriguing. In humans, the perforin allele has extensive single nucleotide variants (SNVs) [18–27]. Clinically, perforin SNVs are viewed unfavorably, as loss of perforin function (activity and/or expression) can result in the lethal childhood disease familial hemophagocytic lymphohistiocytosis type 2 (FHL 2) [27]. However, there are perforin SNVs that do not result in loss of function but rather decrease activity and/or expression [18, 19, 25]. These SNVs do not necessarily result in FHL 2 or other perforinopathies [28–32]. The significance of these perforin SNVs is unknown. However, diversity in immune-related genes has historically been considered advantageous, most notably the genetic diversity in MHC class I molecules [33]. Therefore, if diversity at the perforin allele positively affects inclusive fitness in humans then altered perforin expression will contribute to (1) increase effectiveness at clearing pathogens and/or (2) reduce immune-mediated pathology. To address this hypothesis, one needs to isolate the effect of a single perforin allele on pathogen clearance and immune-mediated pathology. It has recently been documented that perforin-heterozygous mice express approximately one half the perforin messenger RNA (mRNA) and protein of wild-type mice [34, 35]. Perforin-heterozygous mice can therefore be employed to study the effect of a single perforin allele on CNS viral control and immune-mediated BBB disruption.

To better study the critical immune mechanisms of BBB disruption, our laboratory developed the peptide-induced fatal syndrome (PIFS) model [12–15]. In this study, we utilize the PIFS model of CD8 T cell-mediated BBB disruption to address our hypothesis that a single perforin allele can alter the severity of CNS vascular permeability [12–15]. The PIFS model employs Daniel’s strain of Theiler’s murine encephalomyelitis virus (TMEV) as an inducible model of pathologic BBB disruption in C57BL/6 mice [12–15]. Furthermore, since the PIFS model is dependent on H-2D^b^ MHC I haplotype and virus-specific CD8 T cells, this model is a powerful tool in addressing how the effector molecule perforin modulates BBB disruption in a dose-dependent manner [12–15]. In the process, we also address the effect of perforin gene dosage on CNS viral infection.

## Methods

### Animals

Perforin-competent (perforin+/+) and perforin-deficient (perforin−/−) C57BL/6 male mice were obtained from Jackson Laboratory (Bar Harbor, ME; 000664, 002407) at 6 weeks of age. Perforin-heterozygous mice (perforin+/−) were bred at Mayo Clinic Animal Facilities by crossing C57BL/6 perforin+/+ mice with C56BL/6 perforin−/− mice. Mice were used between 6 and 8 weeks of age. All experiments were approved by the Institutional Animal Care and Use Committee of Mayo Clinic.

### TMEV infection

Mice were intracranially (i.c.) injected with 2 × 10^6^ PFU of Daniel’s strain TMEV. Seven days later, at peak CNS inflammation—specifically CD8 T cell expansion—brains were harvested and assessed for immune infiltrate and viral control.

### Lymphocyte isolation from brain and flow cytometry

Flow cytometry was performed as previously described [16, 17]. In brief, whole brains were manually homogenized using a glass dounce in RPMI 1640. Brain suspension was filtered through nylon mesh 100-μm filters into a Percoll and RPMI 1640 solution and spun to isolate a lymphocyte layer. The lymphocyte layer present at the bottom of the solution was diluted, spun, washed, and incubated with the following antibodies for 40 min: D^b^:VP2_121-130_ tetramer-APC, anti-CD45-PerCP, anti-CD4-PE, and anti-NK1.1-FITC followed by a 20-min incubation with anti-CD8a-PeCy7. Following stain, cells were washed with FACS buffer and fixed in 4 % paraformaldehyde (PFA) and 1× PBS (final concentration 2.5 % PFA). One million events were collected per sample. Samples were run on a BD LSRII flow cytometer (BD Biosciences) and then analyzed using FlowJo Software (FlowJo, OR). Three separate experiments were completed to determine CNS immune infiltrate; a representative experiment is shown. Antibodies were purchased from BD Biosciences (San Jose, CA) and Tonbo (San Diego, CA). *n* = 3 perforin−/− mice and *n* = 5 perforin+/− mice and perforin+/+ mice.

### Analysis of viral load

CNS viral control was assessed with real-time PCR (RT-PCR) of the viral genome present in the CNS. RT-PCR was performed as previously described [36]. In brief, whole brain suspension was mixed with TRIzol LS Reagent (Life Technologies) and chloroform. Following incubation, samples were spun down. Aqueous phase was precipitated with isopropanol. RNA was washed using cold 75 % ethanol and resuspended in RNAse-free H_2_O. RNA concentration and quality was determined using a NanoVue Spectrophotometer (GE Healthcare Life Sciences). TMEV RNA was quantified using the one-step QuantiTect SYBR Green Kit for the VP2 gene (forward primer: TGGTCGACTCTGTGGTTACG and reverse primer: GCCGGTCTTGGAAAGATAGT). All data was normalized to mouse β-actin (forward primer: CTGGCACCACACCTTCTACAATGAGCTG and reverse primer: GCACAGCTTCTCTTTGATGTCACGCACGATTTC). RT-PCR was performed on a StepOnePlus Real-Time PCR System (Applied Biosystems) under the following conditions: 50 °C for 2 min and 95 °C for 10 min, followed by 40 cycles of 95 °C for 15 s and 55 °C for 1 min. CT was set just prior to amplification plateau. All samples were normalized to their own mouse actin levels. Gene expression for each sample was calculated using the ΔCT (CT VP2-CT β-actin). Quantification is shown as TMEV gene expression. Three separate experiments were completed to determine CNS viral load; a representative experiment is shown. *n* = 3 perforin−/− mice, *n* = 5 perforin+/− mice, and *n* = 4 perforin+/+ mice.

### Induction of BBB disruption using the PIFS model

CNS vascular permeability was induced as previously described [14–17, 37]. Briefly, all mice were intracranially injected with 2 × 10^6^ PFU Daniel’s strain TMEV. Seven days post infection, at peak CD8 T cell expansion, mice were administered 0.1 mg VP2_121-130_ (FHAGSLLVFM) peptide to induce BBB disruption or mock E7 (RAHYNIVTF) peptide (Genscript), which binds the D^b^ MHC I molecule but is not relevant to TMEV infection [37]. Mice were then monitored hourly for the development of a visual, recognizable deficit.

### MRI acquisition

A Bruker Advance II Tesla 7 vertical bore small animal MRI system (Bruker BioSpin) was used for image acquisition to evaluate CNS vascular permeability. Image acquisition was performed as previously described [38, 39]. In brief, inhalation of anesthesia was induced and maintained during the acquisition using an MRI-compatible monitoring system (Model 1030l; SA Instruments, Stony Brook, NY). Mice were given an intraperitoneal injection of gadolinium using weight dosing of 100 mg/kg, and after a standard delay of 15 min, a volume acquisition T1-weighted spin echo sequence was used (repetition time = 150 ms; echo time = 8 ms; field of view: 32 mm × 19.2 mm × 19.2 mm, matrix: 160 × 96 × 96; number of average = 1) to obtain the T1-weighted images.

### MRI image analysis

Three-dimensional (3D) volumetric analysis was performed as previously described [9, 38, 40, 41]. In brief, the 3D volume of vascular permeability was quantified using Analyze 10 software (Biomedical Imaging Resource Center, Mayo Clinic). The brains from the gadolinium-enhanced T1-weighted images were extracted using the 3D volume extractor tool. The 3D region of interest tool was used to define the areas of gadolinium leakage to generate figures. 3D object rendering, using the volume rendering tool, was performed to visualize the identified volumes of contrast enhancement. Gadolinium leakage volume (measured in voxels) for VP2-treated mice were normalized to the mean of E7 controls of corresponding genotype within an experiment. Data was pooled and is represented on a graph as gadolinium leakage volume normalized to E7 controls ([gadolinium leakage volume VP2 mouse/mean gadolinium leakage volume E7 control of corresponding genotype] = gadolinium leakage volume normalized to E7 controls). *n* = 3 perforin−/− mice; *n* = 6 perforin+/− mice; *n* = 9 perforin+/+ mice treated with VP2_121-130_ peptide.

### Confocal microscopy

Fresh frozen coronal slices from mouse brains were cut on a cryostat and placed onto positively charged slides. Slides were washed twice with PBS and then fixed in 3 % PFA for 15 min. Slides were rinsed three times in PBS, followed by 1-h incubation in 5 % normal goat serum plus 0.5 % Igepal CA-630 (I3021, Sigma-Aldrich) in PBS. Antibodies used for confocal microscopy are as follows: Polyclonal Rabbit Anti-human Fibrinogen (Dako) at a 1:1000 dilution, Monoclonal Mouse Anti-claudin 5 (Invitrogren) at a 1:200 dilution, and Hoechst stain (Sigma) at a 1:1000 dilution.

In order to quantitate the amount of leak into the brain parenchyma, ImageJ software was used to determine the area of claudin-5 staining and fibrinogen staining on 60× images. Then the area of the claudin-5 staining was subtracted from the area of fibrinogen staining (area of leak = area of fibrinogen stain − area of claudin 5 stain). This was done for a minimum of three random sections per mouse. A minimum of three mice were analyzed per genotype. Units are reported in pixels.

### Open field test

To determine if behavioral deficit was present, mice were subjected to an open field test 12–16 h post peptide injection (post-PIFS). Uninfected perforin+/+, perforin+/−, and perforin−/− C57BL/6 mice were used to establish baseline locomotor activity. The open field test was performed as previously described [42]. In brief, following 1-h room habituation, mice were placed in open field chambers (27 cm × 27 cm) equipped with three sets of infrared photobeams to record *X*, *Y*, and *Z* ambulatory movements at a 50-ms resolution (Med Associated, Lafayette, IN), to assess spontaneous locomotor activity. The chambers were located in brightly lit (500 lx), sound-attenuating cubicles. Activity was quantified as horizontal distance traveled (cm) over a series of 60 min. Time spent not making any movements was also measured and is expressed in total resting time in minutes.

### Statistical analysis

Graphs were generated using PRISM software. Data was analyzed in the PRISM software program using a *T* test with Welch’s correction, a one-way ANOVA with Tukey post hoc, or two-way ANOVA. The test is as reported in the figure legends. Significance was considered with a *p* < 0.05. *p* values are reported in the figures and figure legends.

## Results

### Neuroinflammatory profile of perforin-null, perforin-heterozygous, and perforin-WT mice during acute CNS TMEV infection

**Fig. 1:**
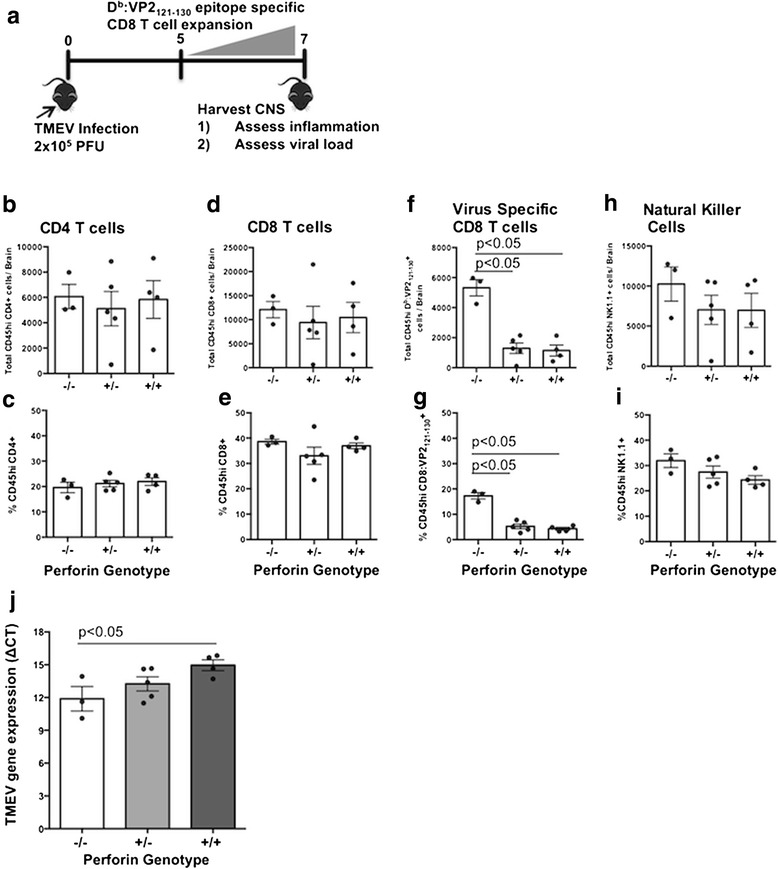
Impact of perforin gene dosage on neuroinflammation and virus load during acute TMEV infection. **a** TMEV-infected perforin−/−, perforin+/−, and perforin+/+ mouse brains were harvested to assess CNS immune profile and viral load. Brain infiltrating lymphocytes were isolated, stained, and analyzed using flow cytometry. Absolute number and frequency as a percentage of CD45hi cells is reported for **b, c** CD4 T cell, **d, e** CD8 T cells, **f, g** virus-specific CD8 T cells, and **h, i** natural killer cells. **j** Whole RNA was also extracted from the brain to assess viral load via qRT-PCR. Mouse groups are as follows: *n* = 3 perforin−/− mice and *n* = 5 perforin+/− mice and perforin+/+ mice for flow cytometry and *n* = 3 perforin−/− mice, *n* = 5 perforin+/− mice, and *n* = 4 perforin+/+ mice for viral control. Three independent experiments were done. A representative experiment is shown. Significance was determined using a one-way ANOVA with a Tukey post hoc test. *p* < 0.05 considered statistically significant

In order to determine if differential perforin expression alters immune cell migration into the CNS, we TMEV-infected perforin-null (perforin−/−), perforin-heterozygous (perforin+/−), and perforin-WT (perforin+/+) C57BL/6 mice. Seven days following infection, the brains were harvested and lymphocytes were isolated through mechanical homogenization followed by Percoll gradient. Frequency and whole number of CD4 T cells, CD8 T cells, natural killer (NK) cells, and antiviral D^b^:VP2_121-130_ epitope-specific CD8 T cells were determined using flow cytometric analysis (Fig. 1). We observed no significant difference between perforin-null, perforin-heterozygous, and perforin-WT mice pertaining to numbers or frequency of CD4 T cells (Fig. 1b, c), CD8 T cells (Fig. 1d, e), or NK cells (Fig. 1h, i) in the brains of mice with a 7-day TMEV infection. However, there was a significant decrease in the number of antiviral D^b^:VP2_121-130_ epitope-specific CD8 T cells in both perforin-heterozygous and perforin-WT mice compared to perforin-null mice (Fig. 1f). This decrease in total D^b^:VP2_121-130_ epitope-specific CD8 T cell number was accompanied by a decreased frequency as a percentage of CD45hi cells (Fig. 1g). Antiviral D^b^:VP2_121-130_ epitope-specific CD8 T cells were the only cells that had a decreased frequency and number in the CNS during a 7-day TMEV infection in wild-type C57BL/6 mice.

### Decreasing perforin expression decreases CNS viral control

Perforin is critical for control of virus infections [28]. Therefore, we next determined the extent to which a single perforin allele would control virus during acute TMEV infection. To determine viral load, total RNA was isolated from 7-day TMEV-infected perforin-null, perforin-heterozygous, and perforin-WT mouse brains. TMEV VP2 gene expression was quantified using RT-PCR and standardized to mouse β-actin [36]. As expected, based on perforin’s antiviral role, perforin-null mice have the highest viral load as determined by quantifying TMEV VP2 gene expression levels 7 days post infection, reflected by the lowest ΔCT values. In contrast, perforin-WT mice have the lowest TMEV VP2 gene expression in the brain. Meanwhile, perforin-heterozygous mice had a trend to have an intermediate TMEV VP2 gene expression level. Perforin-heterozygous mice had a trend to have less TMEV VP2 expression in the brain than the perforin-null mice but more than perforin-WT mice (Fig. 1j). These data demonstrate perforin-heterozygous mice have an intermediate phenotype of CNS viral control. Therefore, perforin alleles are cumulative, with more perforin expression resulting in graded virus control during acute TMEV infection.

### Perforin gene dosage proportionally affects CNS vascular permeability and disorganization of cerebral endothelial cell tight junctions

**Fig. 2:**
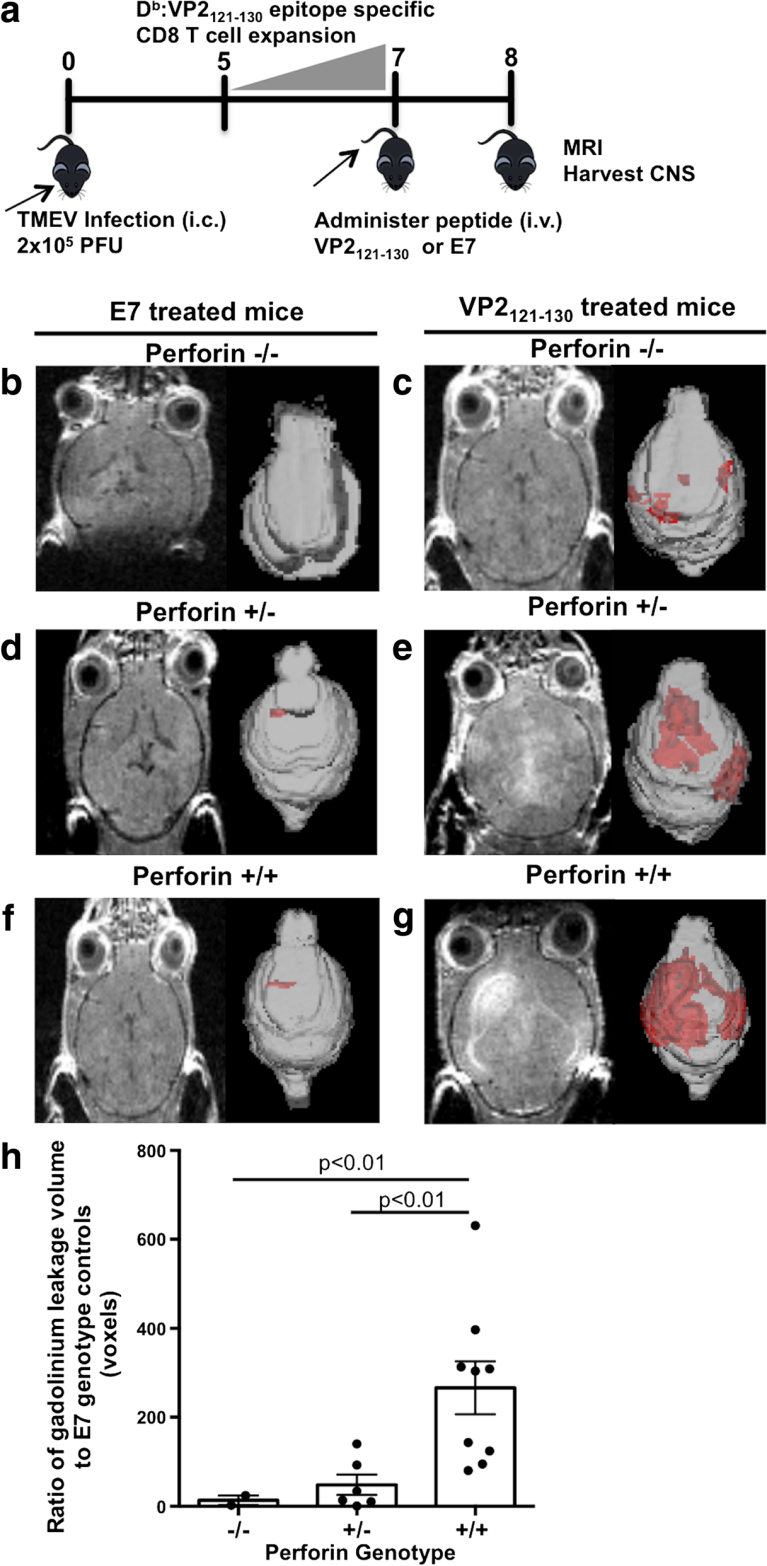
Perforin gene dosage proportionally affects CNS vascular permeability as measured by gadolinium-enhanced T1-weighted MRI. **a** Perforin−/−, perforin+/−, and perforin+/+ mice were evaluated in the PIFS model. On day 8, 12 h post viral peptide, VP2_121-130_, or mock E7 peptide, all mice were subjected to gadolinium-enhanced T1-weighted small animal MRI. **b**–**g** Representative animals are shown for raw MRI image and 3D reconstruction showing the area of gadolinium enhancement in *red*. **h** Gadolinium enhancement was quantified and plotted. The group sizes for VP2_121-130_-exposed peptide mice were as follows: *n* = 3 perforin−/− mice, *n* = 6 perforin+/− mice, and *n* = 9 perforin+/+ mice. All perforin genotypes treated with the VP2_121-130_ peptide were standardized corresponding to the genotype treated with the E7 peptide-treated control (*n* = 3 per group). Significance was determined using Student’s *T* test with Welch’s correction. *p* < 0.05 considered statistically significant

Perforin allele gene dosage did not increase neuroinflammation during acute viral infection. In addition, perforin-null mice exhibit higher viral loads then perforin-heterozygous and perforin-WT mice. We next sought to determine if perforin gene dosage affects BBB disruption. To determine the effect of a single perforin allele on BBB disruption, we employed the peptide-induced fatal syndrome (PIFS) model of CD8 T cell-mediated CNS vascular permeability [14–17, 37]. The PIFS model of immune-mediated BBB disruption is induced through administration of the immunodominant TMEV peptide, VP2_121-130_ [43]. On day 0, C57BL/6 mice are intracranially (i.c.) infected with 2 × 10^6^ PFU Daniel’s strain TMEV. During days 5–7 post infection, there is an expansion of antiviral CD8 T cells specific to the immunodominant epitope D^b^:VP2_121-130_ in the brain [43]. On day 7 of acute TMEV infection, the VP2_121-130_ peptide, or mock E7 peptide, is administered intravenously to mice. This induces BBB disruption which is mediated by D^b^:VP2_121-130_ epitope-specific CD8 T cells. The following day, mice undergo gadolinium-enhanced T1-weighted MRI to assess the extent of CNS vascular permeability [15, 16]. Following MRI, the brains are harvested for histological analysis of cerebral endothelial cell BBB tight junctions (Fig. 2a).

In the PIFS model, CNS vascular permeability initiates at approximately 4 h post administration of the VP2_121-130_ peptide [15]. Meanwhile, behavioral deficit occurs at 12 h post administration of the VP2_121-130_ peptide [15]. Based on this time course, we imaged mice with gadolinium-enhanced T1-weighted MRI to determine the levels of CNS vascular permeability at 12 h, and gadolinium-enhanced lesions were quantified using 3D volumetric analysis and Analyze 12.0 software. Perforin-null mice did not present with gadolinium enhancement following VP2_121-130_ peptide administration (Fig. 2). In contrast, perforin-WT have high gadolinium enhancement (Fig. 2). Meanwhile, perforin-heterozygous mice had an intermediate phenotype pertaining to gadolinium enhancement when compared to perforin-WT mice and perforin-null mice (Fig. 2h).

**Fig. 3:**
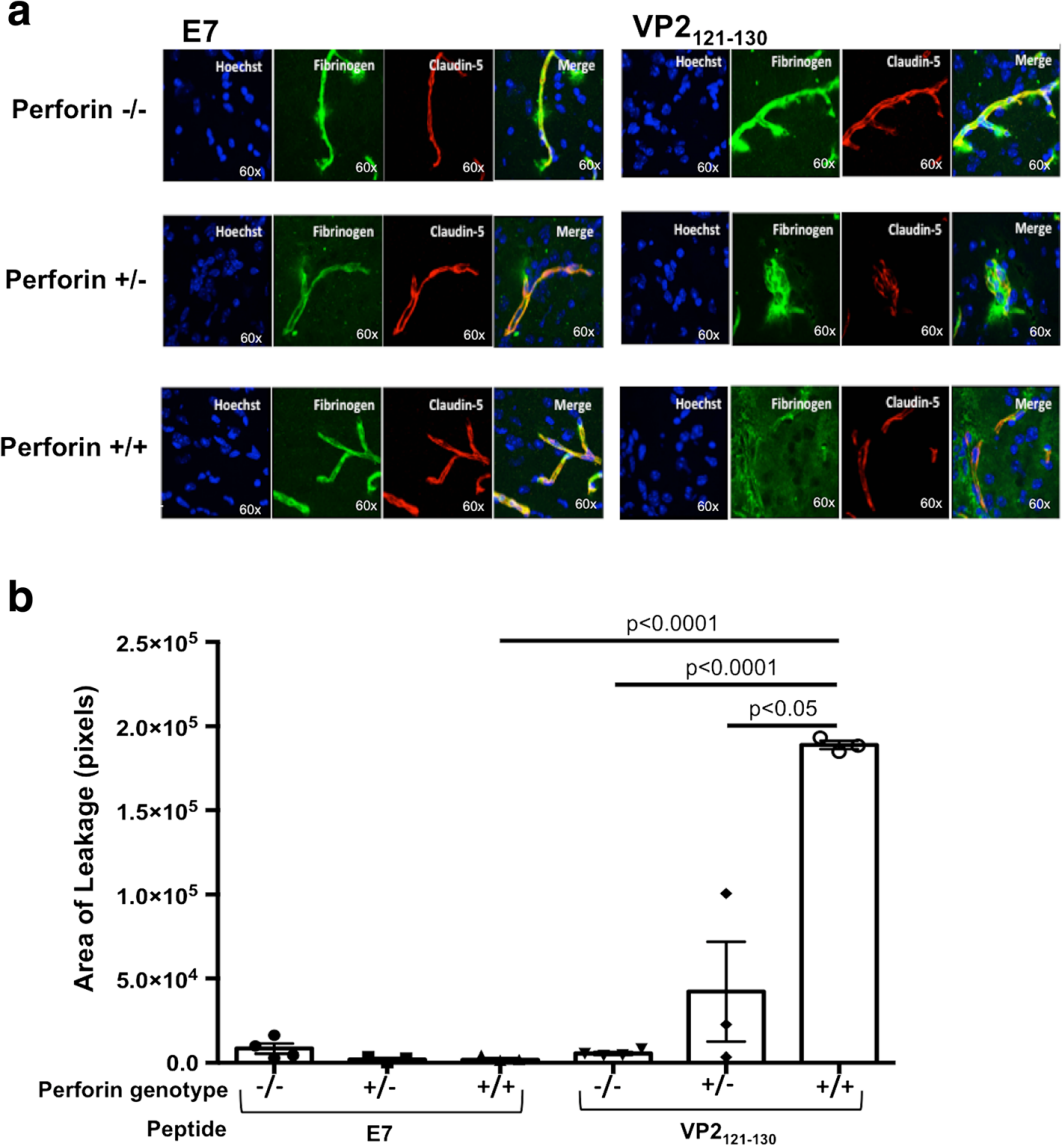
BBB tight junction protein integrity corresponds with perforin gene dosage. Following MRI, brains from perforin−/−, perforin+/−, and perforin+/+ mice that were administered the TMEV antigenic peptide VP2_121-130_ to induce BBB disruption or control peptide E7 were harvested and fresh frozen. **a** Slices were stained for nuclei (Hoechst), blood (fibrinogen), and cerebral endothelial cell tight junction protein (claudin-5), as labeled on the image. Representative 60× images are shown for each genotype (*rows*) and peptide exposure (*columns*). The area of fibrinogen leak was quantified by the following equation: (total area of claudin-5 stain) − (total area of fibrinogen stain) = (total area of leakage). **b** Total area was measured using ImageJ with the units of pixels. The group sizes were as follows: *n* = 4 perforin−/− mice E7 peptide and *n* = 3 for all other genotypes and peptide exposure. Significance was determined using a one-way ANOVA with Tukey post hoc test. *p* < 0.05 considered statistically significant

Post T1 gadolinium-enhanced MRI, perforin-null, perforin-heterozygous, and perforin-WT mouse brains were harvested for immunohistochemistry analysis of cerebral endothelial cell tight junctions and CNS vascular permeability. We observe intact, linear organization of the tight junction protein, claudin-5, on microvessels of BBB-resistant perforin-null mice. Furthermore, fibrinogen staining co-localized with claudin-5, demonstrating that fibrinogen was confined within the microvessel (Fig. 3a). In contrast, perforin-WT mice had more punctate claudin-5 staining, indicative of disorganized tight junctions on microvessels (Fig. 3a). Additionally, we observed extensive fibrinogen leakage into brain parenchyma, indicative of a disrupted BBB in areas of tight junction disorganization (Fig. 3a). Perforin-heterozygous mice did not have as extensive of fibrinogen leakage outside the microvessel as was observed in the perforin-WT mice (Fig. 3a). To quantify CNS vascular leakage, we measured the area of fibrinogen staining outside the microvessel in brain parenchyma. Quantitative analysis of fibrinogen leak was calculated by subtracting the total area of claudin-5 staining from the total area of fibrinogen staining (see the “Methods” section). The results of this analysis were in accordance with our observations using MRI. As shown in Fig. 3, perforin-null mice had negligible fibrinogen leakage outside the microvessel (Fig. 3b). Furthermore, perforin-WT mice have the most extensive fibrinogen leakage outside microvessels (Fig. 3b). Similar to the findings of the MRI study, perforin-heterozygous mice had an intermediate phenotype of BBB disruption and presented with significantly less fibrinogen staining outside of the microvessel. However, leakage was not absent as observed in perforin-null mice (Fig. 3b).

### Expression of a single perforin allele results in locomotor deficit in the PIFS model of immune-mediated BBB disruption

**Fig. 4:**
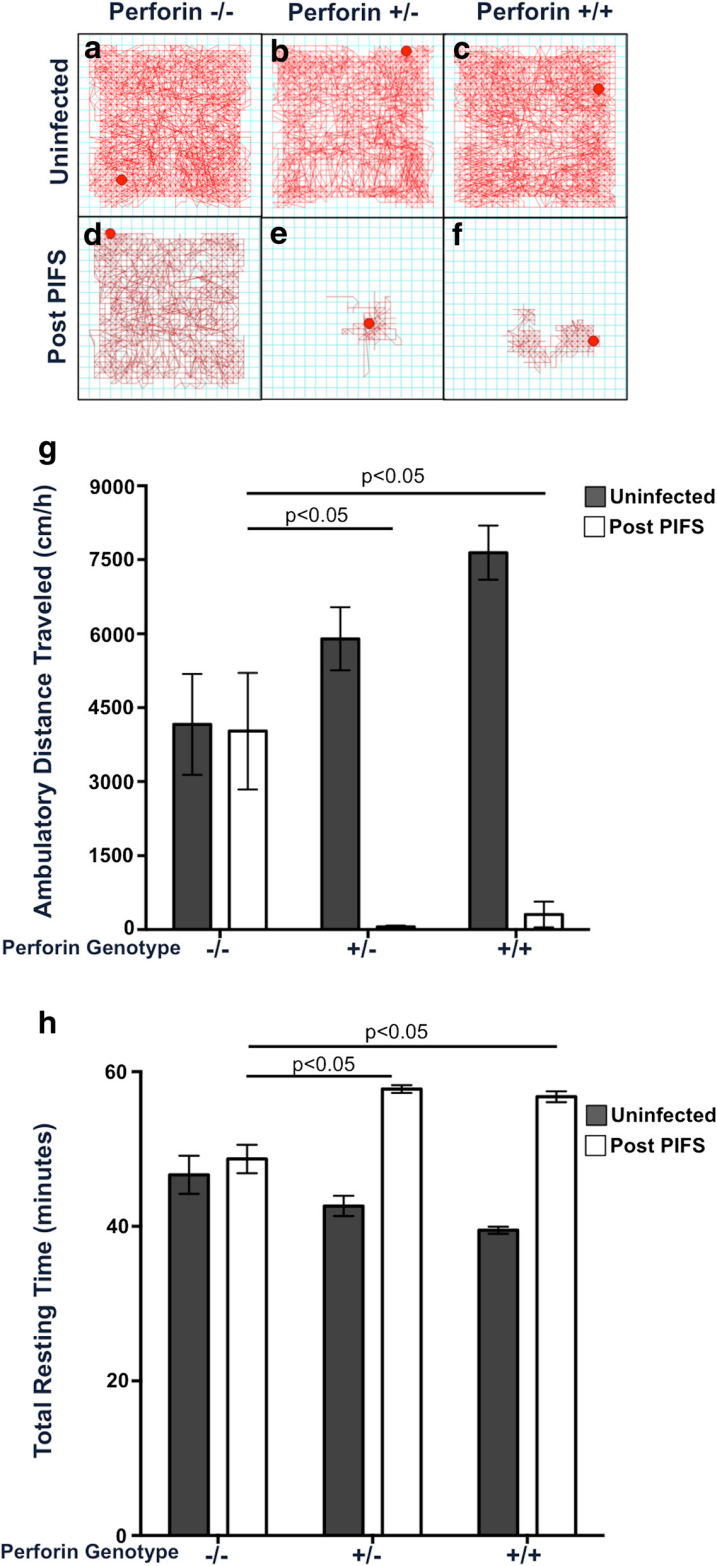
Expression of single perforin allele is sufficient to induce impaired locomotor activity during immune-mediated BBB disruption. Locomotor activity was assessed in the open field behavior test for uninfected or post-PIFS perforin−/−, perforin+/−, and perforin+/+ mice. **a**–**c** Representative activity box tracking images are shown for uninfected **a** perforin−/− mice (*n* = 6), **b** perforin+/− mice (*n* = 8), and **c** perforin+/+ mice (*n* = 5). **d**, **e** Representative activity box tracing images are shown for post-PIFS **d** perforin−/− mice (*n* = 8), **e** perforin+/− mice (*n* = 6), and **f** perforin+/+ mice (*n* = 5). **g** Ambulatory horizontal distance traveled was calculated over 60 min for each genotype for both uninfected (*dark bars*) and post-PIFS (*white bars*). **h** The total resting units, time spent with no movement at all, was also calculated over 60 min for each genotype for both uninfected (*dark bars*) and post-PIFS (*white bars*). Significance was determined using a two-way ANOVA with Tukey post hoc test. *p* < 0.05 considered statistically significant

The intermediate phenotype of CNS permeability observed in perforin-heterozygous mice prompted us to define the effect of a single perforin allele on behavioral deficit during BBB disruption. First, we established there was similar spontaneous locomotor activity and resting time between perforin-null, perforin-heterozygous, and perforin-WT mice (Fig. 4a–c, g, h). To evaluate the effect of a single perforin allele on behavioral deficit during BBB disruption, perforin-null, perforin-heterozygous, and perforin-WT mice were induced to undergo PIFS and locomotor function was evaluated using the open field behavior test at 12 h post administration of the VP2_121-130_ peptide. Perforin-null mice, which are resistant to BBB disruption, had the longest horizontal distance traveled and lowest resting time in the open field test (Fig. 4d, g, h). Perforin-WT mice, which have the highest amount of BBB disruption, had a significant decrease in the horizontal distance traveled and significant increase in total resting units compared to perforin-null mice (Fig. 4f–h). Meanwhile, perforin-heterozygous mice, which have reduced but not abolished BBB disruption, presented with reduced horizontal distance traveled and increased total resting time compared to perforin-null mice. We did not observe a significant difference between perforin-heterozygous and perforin-WT mice in the horizontal distance traveled or total resting units (Fig. 4e, g, h). These data demonstrate a single allele of perforin is sufficient to induce locomotor deficit in the PIFS model.

## Discussion

In this study, we demonstrate the impact of a single perforin allele on BBB disruption and behavioral deficit using the PIFS model of immune-mediated CNS vascular permeability. Our results demonstrate that decreased, but not abolished, perforin expression reduces BBB disruption. This occurred in the absence of increased CNS immune infiltrating cells during acute CNS viral infection. These results support the hypothesis that human perforin SNVs may directly affect pathogen clearance as well as modulate BBB disruption.

Complete abrogation of perforin expression and/or function in human patients results in a rare, lethal, pediatric disease, familial hemophagocytic lymphohistiocytosis type 2 (FHL 2) [27]. Typically, perforin deficiency is correlated with the onset of other diseases coined perforinopathies [28–30, 44]. However, there is a subset of individuals that have decreased perforin expression and activity but live otherwise healthy lives [24–26]. This is most likely due to the perforin allele having extensive SNVs [18–24, 27, 45]. Therefore, to better understand how a single perforin allele contributes to viral clearance during TMEV infection, and CD8 T cell-mediated BBB disruption, we employed the PIFS model. In the PIFS model, perforin-null mice are resistant to pathologic BBB disruption [15]. Given the stark contrast in phenotypes displayed between perforin-null mice and perforin-WT mice, we therefore studied the effect a single perforin allele had on neuropathology. Using perforin-heterozygous mice, which have previously been shown to express approximately half the amount of perforin mRNA and protein as perforin-WT mice, we evaluated the gene dosage effect of perforin on pathogen-associated BBB disruption [34].

We determined the presence of perforin does not increase the frequency or magnitude of brain infiltrating immune cells during acute TMEV infection. There were no differences in CD4 T cell, CD8 T cell, and natural killer cell numbers or frequencies (Fig. 1). We did observe a decrease in the whole number and frequency of D^b^:VP2_121-130_ epitope-specific CD8 T cells in both perforin-heterozygous and perforin-WT mice as compared to perforin-null mice. This observation is in accordance with ongoing hypotheses regarding perforin-mediated immunoregulatory mechanisms previously described by others [46, 47]. In these studies, perforin is defined as having a negative feedback loop to antigen presenting dendritic cells and the cytotoxic CD8 T cell [46]. However, this potential mechanism must be studied further in our model. Despite the decrease in virus-specific CD8 T cells in perforin bearing mice, perforin-WT mice have the highest level of BBB disruption. This demonstrates that BBB disruption is not the results of increased infiltration of activated CD8 T cells.

We next determined how CNS viral control was affected by decreasing perforin amount. As expected, perforin-null mice had the highest amount of relative viral RNA present in the CNS (Fig. 1g). Perforin-WT mice had the least amount of viral RNA in the CNS, suggesting the highest amount of viral control between the three phenotypes (Fig. 1g). Perforin-heterozygous mice had an intermediate level of viral RNA compared to perforin-null and perforin-WT mice. Although perforin does have a dose-dependent effect on CNS viral control during an acute TMEV infection, this does not rule out other antiviral mechanisms being used to control TMEV. Furthermore, CNS viral load and CNS vascular permeability is inversely correlated (Figs. 1 and 2). This experiment demonstrates that BBB disruption was not the results of heightened virus infection.

After establishing that CD8 T cells did not increase with higher perforin expression levels, we sought to determine how perforin gene dosage would affect BBB disruption. Perforin−/− mice were resistant to CNS vascular permeability, as measured by gadolinium-enhanced T1-weighted MRI (Fig. 2b, c, h). In contrast, perforin-WT mice were highly susceptible to BBB disruption as indicated by having approximately 350-fold-more gadolinium leakage when treated with the viral peptide VP2_121-130_ as compared to mice treated with the control peptide E7 (Fig. 2f–h). Perforin-heterozygous mice had an intermediate phenotype. Therefore, decreasing perforin mRNA and protein expression by approximately half resulted in significantly less permeability than perforin-WT mice. Yet, perforin-heterozygous mice were not fully resistant to BBB disruption like the perforin-null mice (Fig. 2d, e, h). Importantly, the change in BBB disruption cannot be attributed to change in CNS inflammation or virus load.

To better define BBB disruption, we investigated the organization of cerebral endothelial cell tight junctions. Perforin-heterozygous mice exhibited an intermediate phenotype in the organization of the cerebral endothelial cells, exhibiting reduced fibrinogen leakage as compared to perforin-WT mice. Furthermore, the perforin-heterozygous mice had higher variability in fibrinogen leak compared to each of the other genotypes. These findings recapitulate the results obtained using MRI. Based on both MRI and confocal data results, we demonstrate BBB disruption is controlled in a perforin gene-dosage-dependent manner. Therefore, it is advantageous, in terms of reducing BBB disruption, to have decreased perforin expression.

We next addressed how in CNS vascular permeability affected behavior. Using the open field test, we were able to measure the extent of motor deficit in these mice. Mice with behavioral deficit will not travel as far and will remain still for longer periods of time as compared to healthy mice. Perforin-null mice experienced no BBB disruption and presented with normal locomotor activity (Fig. 4). When perforin-heterozygous and perforin-WT mice were subjected to the open field test, we discovered that only one functioning perforin allele was required for the development of behavioral deficit (Fig. 4). Despite having a significantly lower level of CNS permeability and more tight junction organization, it remains possible that the extent of BBB disruption perforin-heterozygous mice experienced was enough to induce behavioral deficit. There are several possible reasons perforin-heterozygous mice presented with behavioral deficit, despite the significant decrease in CNS vascular permeability. First, although there is a reduced perforin expression, significant CNS vascular permeability occurred in vital regions of the brain at a sufficient level to induce locomotor deficit. Specifically, perforin-heterozygous mice have significant permeability in the thalamus (data not shown). Permeability in this brain region could account for the locomotor deficit observed. Another possibility is that although perforin-heterozygous mice have reduced BBB disruption, significant CNS vascular permeability was retained. These low levels of permeability could still affect neurochemistry and associated neurological deficit.

One limitation of our model is that we only reduce perforin expression by 50 %. In patients with FHL 2 that receive bone marrow transplants, perforin only needs to reach 10–30 % of normal expression and activity to be curative [48]. Based on these studies, it is presumed that individuals who have 10–30 % perforin expression may not have symptoms associated with FHL 2 or other perforinopathies. However, due to the lack of data, this claim cannot by fully supported. It is possible that greater reduction of perforin, perhaps in the 10–30 % expression range, would decrease CNS vascular permeability to a level that would not result in behavioral deficit but still be expressed at a level that controls a CNS viral infection.

Furthermore, although total perforin expression was reduced, full activity of the monomeric perforin protein is retained in this model. Currently, there are numerous perforin SNVs reported in the literature that affect the activity of this molecule [16–25]. The most notable of these SNVs is 272C>T resulting in an alanine to valine mutation in the amino acid at position 91 (A91V). Currently, it is reported that 5–20 % of the Caucasian population has this mutation which results in a 50 % decrease in perforin activity when homozygous [26]. The consequence of humans harboring perforin SNVs, resulting in reduced activity, remains to be defined. It is possible that the activity of perforin is more critical for maintaining behavioral function than expression alone. Determining how activity altering perforin SNVs directly affect BBB disruption and CNS viral control is therefore of high importance and will continue to be a focus of investigation.

## Conclusions

In conclusion, this paper further defines the underlying mechanism of perforin-mediated BBB disruption. Our data demonstrate BBB disruption is regulated in a perforin dose-dependent manner. Furthermore, one fully active allele of perforin is sufficient to cause behavioral deficit during neuroinflammation-induced BBB disruption. Future studies should focus on altered perforin activity in the context of BBB disruption to better understand how perforin diversity modulates human neurologic disease. Nevertheless, this study demonstrates a potentially advantageous role for decreased perforin expression in reducing BBB disruption. This study also provides insight into the effect SNVs present in a single perforin allele could affect functional deficit in neurological disease.

## Abbreviations

BBB: Blood-brain barrier
CM: Cerebral malaria
CNS: Central nervous system
FHL 2: Familial hemophagocytic lymphohistiocytosis type 2
PFA: Paraformaldehyde
PIFS: Peptide-induced fatal syndrome
SNV: Single nucleotide variant
TMEV: Theiler’s murine encephalomyelitis virus
VHF: Viral hemorrhagic fever
